# Relations between ordinary energy and energy of a self-loop graph

**DOI:** 10.1016/j.heliyon.2024.e27756

**Published:** 2024-03-08

**Authors:** B.R. Rakshith, Kinkar Chandra Das, B.J. Manjunatha, Yilun Shang

**Affiliations:** a Department of Mathematics, Manipal Institute of Technology, Manipal Academy of Higher Education, Manipal 576 104, India; b Department of Mathematics, Sungkyunkwan University, Suwon 16419, Republic of Korea; c Department of Mathematics, Sri Jayachamarajendra College of Engineering, JSS Science and Technology University, Mysuru–570 006, India; d Department of Mathematics, Vidyavardhaka College of Engineering, Mysuru-570 002, Affiliated to Visvesvaraya Technological University, Belagavi-590 018, India; e Department of Computer and Information Sciences, Northumbria University, Newcastle NE1 8ST, UK

**Keywords:** 05C50, Graphs with self-loops, Energy of a graph, Singular values

## Abstract

Let *G* be a graph on *n* vertices with vertex set V(G) and let S⊆V(G) with |S|=α. Denote by $G_{S}$, the graph obtained from *G* by adding a self-loop at each of the vertices in *S*. In this note, we first give an upper bound and a lower bound for the energy of $G_{S}$ ($E(G_{S})$) in terms of ordinary energy (E(G)), order (*n*) and number of self-loops (*α*). Recently, it is proved that for a bipartite graph $G_{S}$, $E(G_{S})\geq E(G)$. Here we show that this inequality is strict for an unbalanced bipartite graph $G_{S}$ with 0<α<n. In other words, we show that there exits no unbalanced bipartite graph $G_{S}$ with 0<α<n and $E(G_{S})=E(G)$.

## 1 Introduction

Let *G* be a simple graph on *n* vertices with vertex set $V(G)=\{v_{1},v_{2},\ldots ,v_{n}\}$. Let S⊆V(G) with |S|=α. The graph $G_{S}$ is obtained from *G* by adding a self-loop at each of the vertices in *S*. Adjacency matrix of $G_{S}$ is denoted by $A(G_{S})$ and is defined as $A(G_{S})=A(G)+D_{S}(G)$, where A(G) is the well-known adjacency matrix of *G* and $D_{S}(G)$ is the diagonal matrix with its *i*-th diagonal entry equal to 1 or 0 according as $v_{i}\in S$ or $v_{i}\notin S$, respectively.

We denote the *i*-th largest eigenvalue of $A(G_{S})$ (resp. A(G)) by $\lambda_{i}^{S}$ (resp. $\lambda_{i}$). The sum of absolute values of $\lambda_{i}$ for i=1,2,…,n is called the energy of a graph *G*. Nowadays it is called as ordinary energy. For its basic mathematical properties, including various lower and upper bounds, see [1], [3], [4], [5], [7] and especially the book [11]. Applications of graph energy can be found in the articles [13], [14]. The sum $\sum_{i=1}^{n}\left|\lambda_{i}^{S}-\frac{\alpha }{n}\right|$ is called the energy of graph $G_{S}$ and is denoted by $E(G_{S})$. The definition of energy of a graph with self-loops was put forward very recently by Gutman et al. in [8]. The authors in [8] showed that if *G* is a bipartite graph, then $E(G_{S})=E(G_{V(G)﹨S})$. Also, they conjectured that $E(G_{S})>E(G)$ for 1≤α≤n−1. However this conjecture was disproved in [10] by means of counterexamples. In [2], Akbari et al. showed that this conjecture was nevertheless not a complete miss by proving that energy of a bipartite graph $G_{S}$ is always greater than or equal to its ordinary energy. In [12], Popat et al. obtained a family of graphs which satisfies the property $E(G_{S})=E(G)$ and 0<α<n. Some bounds on energy of self-loop graph are presented in [2], [8]

Motivated by this, in this paper, we give an upper bound and a lower bound for the energy of $G_{S}$ ($E(G_{S})$) in terms of ordinary energy (E(G)), order (*n*) and number of self-loops (*α*). Also, we show that for an unbalanced bipartite graph $G_{S}$ with 0<α<n, $E(G_{S})>E(G)$. In other words, we show that there exits no unbalanced bipartite self-loops graph $G_{S}$ with 0<α<n and $E(G_{S})=E(G)$.

## 2 Main results

Let *A* be an n×n complex matrix and denote its singular values by $s_{1}(A)\geq s_{2}(A)\geq \cdots \geq s_{n}(A)$. Since the matrices A(G) and $A(G_{S})$ are symmetric, $E(G)=\sum_{i=1}^{n}s_{i}(A(G))$ and $E(G_{S})=\sum_{i=1}^{n}s_{i}\left(A(G_{S})-\frac{\alpha }{n}I_{n}\right)$. We need the following lemmas to prove our main result.

Lemma 2.1 [6]*Let A and B be two*n×n*matrices. Then*

$$\sum_{i=1}^{n}s_{i}(A+B)\leq \sum_{i=1}^{n}s_{i}(A)+\sum_{i=1}^{n}s_{i}(B)$$

*and the equality holds if and only if there exists an unitary matrix P such that both the matrices PA and PB are positive semi-definite. Moreover, if A and B are real matrices, then the matrix P can be taken as real orthogonal.*

Lemma 2.2 [9]*Let*$A={\left[a_{ij}\right]}_{n\times n}$*be a positive semi-definite matrix such that*$a_{ii}=0$*for some*1≤i≤n*. Then*$a_{ij}=a_{ji}=0$*for all*1≤j≤n*.*

The following lemma is the polar decomposition of a matrix.

Lemma 2.3 [9]*Let A be a real square matrix of order n. Then there exists an orthogonal matrix P and a positive definite matrix Q such that*A=PQ*. Moreover the matrix Q is uniquely determined by A as*$Q=\sqrt{A^{T}A}$*.*

Lemma 2.4 [9]*Let A a real matrix of order*m×n*and let*q=min{m,n}*. If*$\sigma_{1}\geq \sigma_{2}\geq \cdots \geq \sigma_{q}$*are the singular values of A, then the eigenvalues of the matrix*$\left[\begin{array}{cc} \text{0}\; & A\\ A^{T}\; & \text{0} \end{array}\right]$*are*$-\sigma_{1}\leq -\sigma_{2}\leq \cdots \leq -\sigma_{q}\leq \underset{\left|n-m\right|}{\underset{︸}{0=0=\cdots =0}}\leq \sigma_{q}\leq \sigma_{q-1}\leq \cdots \leq \sigma_{1}$*.*

We denote the complete graph on *n* vertices by $K_{n}$ and its complement graph by ${\bar{K}}_{n}$ or $nK_{1}$. The following theorem gives an upper bound and a lower bound for $E(G_{S})$ in terms of E(G), *n* and *α*.

Theorem 2.5 *Let G be a graph on n vertices and let*S⊆V(G)*with*|S|=α*. Then*$E(G)-\frac{2\alpha (n-\alpha )}{n}\leq E(G_{S})\leq E(G)+\frac{2\alpha (n-\alpha )}{n}$*. Moreover the right inequality holds if and only if*α=0,n*or*$G≅nK_{1}$*. Whereas the left inequality holds if and only if*α=0,n*.*

Proof Let G[S] and G[V(G)﹨S] be the subgraphs of *G* induced by the vertex sets *S* and V(G)﹨S, respectively. Then

$$A(G)=\left[\begin{array}{cc} A(G[S])\; & X\\ X^{T}\; & A(G[V(G)﹨S]) \end{array}\right]\;\;\text{and}\;\;A(G_{S})=\left[\begin{array}{cc} A(G[S])+I_{\alpha }\; & X\\ X^{T}\; & A(G[V(G)﹨S]) \end{array}\right],$$

where *X* is a (0,1)-matrix.

Right inequality: We have

(1) $$A(G_{S})-\frac{\alpha }{n}I_{n}=A(G)+\left[\begin{array}{cc} \left(1-\frac{\alpha }{n}\right)I_{\alpha }\; & \text{0}\\ \text{0}\; & -\frac{\alpha }{n}I_{n-\alpha } \end{array}\right].$$

Applying Lemma 2.1 to equation (1), we get

(2) $$\sum_{i=1}^{n}s_{i}\left(A(G_{S})-\frac{\alpha }{n}I_{n}\right)\leq \sum_{i=1}^{n}s_{i}(A(G))+\sum_{i=1}^{n}s_{i}\left(\left[\begin{array}{cc} \left(1-\frac{\alpha }{n}\right)I_{\alpha }\; & \text{0}\\ \text{0}\; & -\frac{\alpha }{n}I_{n-\alpha } \end{array}\right]\right).$$

That is,

(3) $$E(G_{S})\leq E(G)+\frac{2\alpha (n-\alpha )}{n}.$$

Proving the right equality. Suppose the equality in (3) holds. Then the equality in (2) also holds and thus by Lemma 2.1 there exists an orthogonal matrix *P* such that the matrices PA(G) and $P\left[\begin{array}{cc} \left(1-\frac{\alpha }{n}\right)I_{\alpha }\; & \text{0}\\ \text{0}\; & -\frac{\alpha }{n}I_{n-\alpha } \end{array}\right]$ are positive semi-definite. Moreover, since A(G) is symmetric, we can further assume that *P* is symmetric. Let $P=\left[\begin{array}{cc} P_{11}\; & P_{12}\\ P_{21}\; & P_{22} \end{array}\right]$ and 0<α<n. Since $P\left[\begin{array}{cc} \left(1-\frac{\alpha }{n}\right)I_{\alpha }\; & \text{0}\\ \text{0}\; & -\frac{\alpha }{n}I_{n-\alpha } \end{array}\right]$ and *P* are symmetric matrices, we must have

$$\left[\begin{array}{cc} \left(1-\frac{\alpha }{n}\right)P_{11}\; & -\frac{\alpha }{n}P_{12}\\ \;\\ \left(1-\frac{\alpha }{n}\right)P_{21}\; & -\frac{\alpha }{n}P_{22} \end{array}\right]=\left[\begin{array}{cc} \left(1-\frac{\alpha }{n}\right)P_{11}\; & \left(1-\frac{\alpha }{n}\right)P_{12}\\ \;\\ -\frac{\alpha }{n}P_{21}\; & -\frac{\alpha }{n}P_{22} \end{array}\right].$$

Therefore $P_{12}=P_{21}=\text{0}$, otherwise $1-\frac{\alpha }{n}=-\frac{\alpha }{n}$, that is, 1=0, a contradiction. Thus $P=\left[\begin{array}{cc} P_{11}\; & \text{0}\\ \text{0}\; & P_{22} \end{array}\right]$, where $P_{11}$ and $P_{22}$ are orthogonal matrices of order *α* and n−α. Now, $P\left[\begin{array}{cc} \left(1-\frac{\alpha }{n}\right)I_{\alpha }\; & \text{0}\\ \text{0}\; & -\frac{\alpha }{n}I_{n-\alpha } \end{array}\right]$ is positive semi-definite and so $P_{11}$ is positive semi-definite and $P_{22}$ is negative semi-definite. As, $P_{11}^{2}=I_{\alpha }$, $P_{22}^{2}=I_{n-\alpha }$, $P_{11}$ is a positive semi-definite matrix and $P_{22}$ is negative semi-definite matrix, the minimal polynomial equation of $P_{11}$ and $P_{22}$ must be x−1=0 and x+1=0, respectively. Therefore, $P_{11}=I_{\alpha }$ and $P_{22}=-I_{n-\alpha }$. Hence, $P=\left[\begin{array}{cc} I_{\alpha }\; & \text{0}\\ \text{0}\; & -I_{n-\alpha } \end{array}\right]$. Now, $PA(G)=\left[\begin{array}{cc} A(G[S])\; & X\\ -X^{T}\; & -A(G[V(G)﹨S]) \end{array}\right]$ and since PA(G) is positive semi-definite with all its diagonal entries equal to 0, by Lemma 2.2, we must have A(G)=0. So, $G≅nK_{1}$. Conversely, if α=0,n or $G≅nK_{1}$, then one can easily see that equality holds.

Left inequality: From (1) and by Lemma 2.1, we get

(4) $$\sum_{i=1}^{n}s_{i}\left(A(G)\right)\leq \sum_{i=1}^{n}s_{i}\left(A(G_{S})-\frac{\alpha }{n}I_{n}\right)+\sum_{i=1}^{n}s_{i}\left(\left[\begin{array}{cc} \left(\frac{\alpha }{n}-1\right)I_{\alpha }\; & \text{0}\\ \text{0}\; & \frac{\alpha }{n}I_{n-\alpha } \end{array}\right]\right).$$

Therefore,

(5) $$E(G)\leq E(G_{S})+\frac{2\alpha (n-\alpha )}{n}$$

Proving the left equality. Suppose the equality in (5) holds. Then the equality in (4) also holds and thus by Lemma 2.1 there exists an orthogonal matrix *P* such that the matrices $P\left(A(G_{S})-\frac{\alpha }{n}I_{n}\right)$ and $P\left[\begin{array}{cc} \left(\frac{\alpha }{n}-1\right)I_{\alpha }\; & \text{0}\\ \text{0}\; & \frac{\alpha }{n}I_{n-\alpha } \end{array}\right]$ are positive semi-definite. Assume that 0<α<n. Let $P=\left[\begin{array}{cc} P_{11}\; & P_{12}\\ P_{21}\; & P_{22} \end{array}\right]$. By the similar argument as above, we get $P_{12}=P_{21}=\text{0}$, $P_{11}=-I_{\alpha }$ and $P_{22}=I_{n-\alpha }$. Therefore,

$$P\left(A(G_{S})-\frac{\alpha }{n}I_{n}\right)=\left[\begin{array}{cc} -A(G[S])+\left(\frac{\alpha }{n}-1\right)I_{\alpha }\; & -X\\ X^{T}\; & A(G[V(G)﹨S])-\frac{\alpha }{n}I_{n-\alpha } \end{array}\right],$$

and its diagonal entries are negative for 0<α<n. This implies that $P(A(G_{S})-\frac{\alpha }{n}I_{n})$ is not positive semi-definite, a contradiction. Thus α=0 or *n*. Conversely, if α=0,n, then one can easily see that equality holds. This completes the proof. □

The following corollary follows immediately from the above theorem.

Corollary 2.6 *Let G be a graph on n vertices. Then for any vertex v of G, we have*

$$E(G)-2<E(G_{\left\{v\right\}})<E(G)+2.$$

Theorem 2.7 *Let G be an unbalanced bipartite graph on n vertices and let* S⊆V(G) *with* |S|=α *. Then* $E(G)<E(G_{S})$ *for* 0<α<n *.*

Proof Let *U* and *W* be the vertex partition sets of the bipartite graph *G* such that $|U|=n_{1}$ and $|W|=n_{2}$, where $n=n_{1}+n_{2}$. Suppose $S_{1}=S\cap U$ and $S_{2}=S\cap W$. Then

$$A(G)=\left[\begin{array}{cc} 0\; & X\\ X^{T}\; & 0 \end{array}\right]\;\text{and}\;A(G_{S})=\left[\begin{array}{cc} A({\left[n_{1}K_{1}\right]}_{S_{1}})\; & X\\ X^{T}\; & A({\left[n_{2}K_{1}\right]}_{S_{2}}) \end{array}\right],$$

where *X* is a (0, 1)-matrix of order $n_{1}\times n_{2}$.

Let $A^{\prime }(G_{S})=\left[\begin{array}{cc} -A({\left[n_{1}K_{1}\right]}_{S_{1}})\; & X\\ X^{T}\; & -A({\left[n_{2}K_{1}\right]}_{S_{2}}) \end{array}\right]$. Then

(6) $$\left(A(G_{S})-\frac{\alpha }{n}I_{n}\right)+\left(A^{\prime }(G_{S})+\frac{\alpha }{n}I_{n}\right)=2A(G).$$

Applying Lemma 2.1 to the equation (6), we get

(7) $$\sum_{i=1}^{n}s_{i}\left(A(G_{S})-\frac{\alpha }{n}I_{n}\right)+\sum_{i=1}^{n}s_{i}\left(A^{\prime }\left(G_{S}\right)+\frac{\alpha }{n}I_{n}\right)\geq 2\sum_{i=1}^{n}s_{i}(A(G)).$$

Since $A(G_{S})-\frac{\alpha }{n}I_{n}=\left[\begin{array}{cc} I_{n_{1}}\; & \text{0}\\ \text{0}\; & -I_{n_{2}} \end{array}\right]\left[-A^{\prime }(G_{S})-\frac{\alpha }{n}I_{n}\right]\left[\begin{array}{cc} I_{n_{1}}\; & \text{0}\\ \text{0}\; & -I_{n_{2}} \end{array}\right]$, the matrices $A(G_{S})-\frac{\alpha }{n}I_{n}$ and $-\left[A^{\prime }(G_{S})+\frac{\alpha }{n}I_{n}\right]$ are similar. Therefore these matrices share the same singular eigenvalues. Thus from equation (7), we have

(8) $$E(G_{s})\geq E(G)$$

Suppose that the equality in relation (8) holds. Then the equality in relation (7) holds and thus by Lemma 2.1 there exists a real orthogonal matrix *P* such that the matrices $P\left(A(G_{S})-\frac{\alpha }{n}I_{n}\right)$ and $P\left(A^{\prime }(G_{S})+\frac{\alpha }{n}I_{n}\right)$ are positive semi-definite. Now by Lemma 2.3,

(9) $$P\left(A(G_{S})-\frac{\alpha }{n}I_{n}\right)=\sqrt{{\left(A(G_{S})-\frac{\alpha }{n}I_{n}\right)}^{T}\left(A(G_{S})-\frac{\alpha }{n}I_{n}\right)}$$

and

(10) $$P\left(A^{\prime }(G_{S})+\frac{\alpha }{n}I_{n}\right)=\sqrt{{\left(A^{\prime }(G_{S})+\frac{\alpha }{n}I_{n}\right)}^{T}\left(A^{\prime }(G_{S})+\frac{\alpha }{n}I_{n}\right)}.$$

Since the matrices $A(G_{S})-\frac{\alpha }{n}I_{n}$ and $-\left[A^{\prime }(G_{S})+\frac{\alpha }{n}I_{n}\right]$ are similar, from equations (9) and (10) it follows that the matrices $P\left(A(G_{S})-\frac{\alpha }{n}I_{n}\right)$ and $P\left(A^{\prime }(G_{S})+\frac{\alpha }{n}I_{n}\right)$ are similar. In particular,

(11) $$P\left(A(G_{S})-\frac{\alpha }{n}I_{n}\right)=\left[\begin{array}{cc} I_{n_{1}}\; & \text{0}\\ \text{0}\; & -I_{n_{2}} \end{array}\right]\left[P\left(A^{\prime }(G_{S})+\frac{\alpha }{n}I_{n}\right)\right]\left[\begin{array}{cc} I_{n_{1}}\; & \text{0}\\ \text{0}\; & -I_{n_{2}} \end{array}\right]$$

Let $P=\left[\begin{array}{cc} P_{11}\; & P_{12}\\ P_{21}\; & P_{22} \end{array}\right]$, where $P_{11}$ and $P_{22}$ are square matrices of order $n_{1}$ and $n_{2}$. Then from equation (11), we get

$$\left[\begin{array}{cc} P_{11}\left[A({\left[n_{1}K_{1}\right]}_{S_{1}})-\frac{\alpha }{n}I_{n_{1}}\right]+P_{12}X^{T}\; & P_{11}X+P_{12}\left[A({\left[n_{2}K_{1}\right]}_{S_{2}})-\frac{\alpha }{n}I_{n_{2}}\right]\\ \;\\ P_{21}\left[A({\left[n_{1}K_{1}\right]}_{S_{1}})-\frac{\alpha }{n}I_{n_{1}}\right]+P_{22}X^{T}\; & P_{21}X+P_{22}\left[A({\left[n_{2}K_{1}\right]}_{S_{2}})-\frac{\alpha }{n}I_{n_{2}}\right] \end{array}\right]$$

$$=\left[\begin{array}{cc} -P_{11}\left[A({\left[n_{1}K_{1}\right]}_{S_{1}})-\frac{\alpha }{n}I_{n_{1}}\right]+P_{12}X^{T}\; & -P_{11}X+P_{12}\left[A({\left[n_{2}K_{1}\right]}_{S_{2}})-\frac{\alpha }{n}I_{n_{2}}\right]\\ \;\\ P_{21}\left[A({\left[n_{1}K_{1}\right]}_{S_{1}})-\frac{\alpha }{n}I_{n_{1}}\right]-P_{22}X^{T}\; & P_{21}X-P_{22}\left[A({\left[n_{2}K_{1}\right]}_{S_{2}})-\frac{\alpha }{n}I_{n_{2}}\right] \end{array}\right].$$

Therefore, $P_{11}\left[A({\left[n_{1}K_{1}\right]}_{S_{1}})-\frac{\alpha }{n}I_{n_{1}}\right]=\text{0}$ and $P_{22}\left[A({\left[n_{2}K_{1}\right]}_{S_{2}})-\frac{\alpha }{n}I_{n_{2}}\right]=\text{0}$. Since 0<α<n, we must have $P_{11}=P_{22}=0$. Thus, $P=\left[\begin{array}{cc} \text{0}\; & P_{12}\\ P_{21}\; & \text{0} \end{array}\right]$. Since $n_{1}\neq n_{2}$, by Lemma 2.4, 0 is an eigenvalue of *P* (an orthogonal matrix), a contradiction. Thus $E(G_{S})>E(G)$. □

## CRediT authorship contribution statement

**B.R. Rakshith:** Writing – review & editing, Writing – original draft, Investigation, Formal analysis. **Kinkar Chandra Das:** Writing – review & editing, Writing – original draft, Investigation, Formal analysis. **B.J. Manjunatha:** Writing – review & editing, Writing – original draft, Investigation, Formal analysis. **Yilun Shang:** Writing – review & editing, Writing – original draft, Investigation, Formal analysis.

## Declaration of Competing Interest

The authors declare the following financial interests/personal relationships which may be considered as potential competing interests: Yilun Shang is a Section Editor for Heliyon.

## Data Availability

No data was used for the research described in the article.

## References

[1] Akbari S., Das K.C., Ghahremani M., Koorepazan-Moftakhar F., Raoufi E. (2021). Energy of graphs containing disjoint cycles. MATCH Commun. Math. Comput. Chem..

[2] Akbari S., Menderj H.A., Ang M.H., Lim J., Ng Z.C. (2023). Some results on spectrum and energy of graphs with loops. Bull. Malays. Math. Sci. Soc..

[3] Das K.C., Alazemi A., Andelić M. (2020). On energy and Laplacian energy of chain graphs. Discrete Appl. Math..

[4] Das K.C., Mojallal S.A., Sun S. (2019). On the sum of the *k* largest eigenvalues of graphs and maximal energy of bipartite graphs. Linear Algebra Appl..

[5] Das K.C., Gutman I. (2016). Bounds for the energy of graphs. Hacet. J. Math. Stat..

[6] Day J., So W. (2007). Singular value inequality and graph energy change. Electron. J. Linear Algebra.

[7] Ganie H.A., Shang Y. (2022). On the spectral radius and energy of signless Laplacian matrix of digraphs. Heliyon.

[8] Gutman I., Redžepović I., Furtula B., Sahal A.M. (2021). Energy of graphs with self-loops. MATCH Commun. Math. Comput. Chem..

[9] Horn R., Johnson C. (1989).

[10] Jovanović I., Zogić E., Glogić E. (2023). On the conjecture related to the energy of graphs with self-loops. MATCH Commun. Math. Comput. Chem..

[11] Li X., Shi Y., Gutman I. (2012).

[12] Popat K.M., Shingal K.R. (2023). Some new results on energy of graphs with self loops. J. Math. Chem..

[13] Redžepović I., Furtula B. (2020). Predictive potential of eigenvalue-based topological molecular descriptors. J. Comput.-Aided Mol. Des..

[14] Tratnik N., Radenković S., Redžepović I., Finšgar M., Žigert Pleteršek P. (2021). Predicting corrosion inhibition effectiveness by molecular descriptors of weighted chemical graphs. Croat. Chem. Acta.

